# Hecogenin a Plant Derived Small Molecule as an Antagonist to BACE-1: A Potential Target for Neurodegenerative Disorders

**DOI:** 10.3390/metabo13060758

**Published:** 2023-06-16

**Authors:** Deepthi Padmanabhan, Manzer H. Siddiqui, Purushothaman Natarajan, Senthilkumar Palanisamy

**Affiliations:** 1 Department of Genetic Engineering, School of Bioengineering, SRM Institute of Science and Technology, Kattankulathur 603203, India; 2 Department of Botany and Microbiology, College of Science, King Saud University, Riyadh 11451, Saudi Arabia; 3 Department of Biology, West Virginia State University, Institute, WV 25112-1000, USA

**Keywords:** phytocompounds, neurodegenerative diseases, plant therapeutics, medicinal plants, drug discovery

## Abstract

The field of drug discovery has recognized the significance of computer-aided drug design. Recent advancements in structure identification and characterization, bio-computational science and molecular biology have significantly contributed to the development of novel treatments for various diseases. Alzheimer’s disease is prevalent in over 50 million affected people, with the pathological condition of amyloidal plaque formation by the beta-amyloidal peptide that results in lesions of the patient’s brain, thus making the target prediction and treatment a hurdle. In this study, we evaluated the potential of 54 bioactive compounds from *Justicia adhatoda L.* and *Sida cordifolia L.* identified through LC-MS/MS against the β-site amyloid precursor cleaving enzyme (beta-secretase) that results in the formation of amyloidal plaques. To study the drug-likeness of the phytocompounds, Lipinski’s rule of five for ADME profiling and toxicity prediction was performed. Molecular docking was performed using auto-dock tool of PyRx software; molecular dynamic simulations were performed using the Schrodinger suite. Molecular docking against BACE-1 protein revealed that hecogenin, identified from *S. cordifolia* has a broad spectrum of pharmacological applications and a binding affinity score of −11.3 kcal/Mol. The Hecogenin–BACE-1 protein complex was found to be stable after 30 ns of MD simulation, resulting in its substantial stability. Further studies focusing on the in vivo neuroprotective activity of hecogenin against the disease will pave the way for efficient drug discovery from natural sources in a precise manner.

## 1. Introduction

Medicinal plants serve as valuable sources of secondary metabolites (SMs), which play a crucial role in the exploration of novel drugs. A wide range of SMs are extracted from medicinal plants such as flavonoids, steroids, terpenoids, tannins, coumarins, quinones and alkaloids. These diverse compounds possess various pharmacological properties. In silico approach has been widely used to study the therapeutic potential by determining its absorption, metabolism, distribution and excretion by minimizing its toxicity [1]. Phytomedicine, an eminent form of traditional medicine, has long been integral to the global therapeutic approach for effectively managing various pathological conditions. It is the most widely accepted form of medicine due to its availability and affordability [2].

*Justicia adhatoda*, an invasive species of the *Acanthaceae* family, is a minuscule shrub that grows up to 3 m heigh. This plant has been used to treat numerous chronic ailments since ancient times [3]. The pharmacological properties exhibited by the plant are wound healing, anti-tubercular, and antipyretic, bronchitis, cough, dysentery and diarrhea [4]. The roots of *Sida cordifolia*, a perennial subshrub belonging to the family *Malvaceae*, have been used to treat various pharmacological disorders since 2000 years ago. The root powder of the plant, when mixed with honey or milk, provides the human system with immunity and treats various infectious diseases. The plant has various ethnopharmacological properties such as anti-inflammatory, ant-microbial, anti-infective, wound healing and is also a potent antidote to snake venom. Many in vivo studies on the crude extract of this plant suggest its strong immunomodulatory effect [5].

Medicinal plants and natural chemicals extracted from these plants have been employed in vivo or clinical experiments to manage neurological conditions and symptoms. Neurodegenerative disorders have been characterized by an increase in reactive oxygen species production in the central nervous system and mitochondrial activity reduction. Most women after menopause reported higher pervasiveness of Alzheimer’s disease than men. To treat such cases, hormone replacement therapy has been used to decrease the risk of AD and decrease the accelerating cognitive function [6,7,8].

Due to the highly complex pathogenesis of Alzheimer’s disease, target prediction and treatment have been a hurdle. The most characterized pathological condition is the formation of amyloidal plaques by the beta-amyloid peptide that results in the lesions of the patient’s brain. The enzyme BACE-1 plays a vital role in beta-amyloid generation. Based on the “amyloid cascade hypothesis”, the pathogenesis of Alzheimer’s disease is the accumulation of amyloidal β neurotoxic oligomers. A study suggests that the knockout of the enzyme results in no neuronal loss or memory defects. Although many body parts contain the beta-secretase enzyme, the highest activity levels are found in neural tissues, which in turn are comparatively most active in the brain neural cells [9]. The molecular docking tool is used to study the inclination of small drug molecule with the protein target to predict its binding affinity and interactions. The tool plays a major role in the ethical design of medicines and is also involved in modern advanced drug testing [10]. The technique predicts the toxicity effects of the compounds. Although many drugs like chloroquine, remediffer and hydroxychloroquine are conventionally tested—and it is found to be therapeutic in laboratory conditions during the pandemic—traditional medicine has been used to treat the disease outbreak with medicinal plants instead of chemical synthesis [11].

This study aimed to determine the anti-neurodegenerative effect of phytochemicals derived from *S. cordifolia* and *J. adhatoda* through LC-MS/MS analysis. The best-docked pose is determined by the number of hydrogen bonds and their binding affinity. The stability of the protein–ligand complex at specific conditions is determined using molecular simulation studies. The *in silico* toxicity profiling of the major metabolites is studied using the SwissADME tool.

## 2. Materials and Methods

### 2.1. Plant Material

Plant materials of *Sida cordifolia* and *Justicia adhathoda* were collected from Potheri, Chengalpattu district, Tamil Nadu, India (GPS coordinates: 12°82′21.5″ N 80°03′72.40″ E). Voucher specimen was deposited at the Madras Christian College Herbarium (https://mcc.edu.in/, accessed on 10 May 2023, Dr. Senthilkumar Umapathy, (senthilumapathy@mcc.edu.in) under the voucher number DP101 (*Sida cordifolia*) and DP102 (*Justicia adhathoda*).

### 2.2. Extraction of Bioactive Compounds from Justicia adhatoda and Sida cordifolia

Fresh leaves were collected dried and pulverized with an electric blender. A total of 10 g of powdered leaf samples were macerated at room temperature for 3 days with methanol and then concentrated using the rotary evaporator. The samples were centrifuged before proceeding for further analysis and stored at −20 °C. The bioactive compounds from *S. cordifolia* were retrieved from previously published data [12].

### 2.3. Antioxidant Profiling of Justicia adhatoda and Sida cordifolia Methanolic Extracts

#### 2.3.1. Total Phenolic Content (TPC)

The total phenolic content present in the methanolic extract of *J. adhatoda* and *S. cordifolia* was determined using Folin’s-Ciocalteu reagent (FC). To 1 ml of extract, 0.5 mL of FC reagent was added. TPC was expressed as mg gallic acid equivalents per gram of the extract (mg GAE/g of extract) using gallic acid as a standard (1 mg/mL) [13].

#### 2.3.2. Total Flavonoid Content

To 0.5 mL of the plant extract, 0.5 mL of 2% aluminum chloride was added and the final volume was made up to 10 mL with distilled water and incubated at room temperature for 1 h. The absorbance was read at 420 nm with a Shimadzu UV-Vis spectrophotometer. Quercetin was used as a standard reference (1 mg/mL) [14].

#### 2.3.3. 2, 2-Diphenyl-1-picrylhydrazyl (DPPH) Assay

Ascorbic acid at a concentration of 1 mg/mL was used as a standard to determine the antioxidant profile of *J. adhatoda* and *S. cordifolia.* To 20 µL of plant extract, 150 µL of DPPH solution was added and the final volume was made up to 200 µL with methanol. Incubated at room temperature in the dark for 30 min and absorbance was read at 517 nm in a spectrophotometer [15].

#### 2.3.4. FRAP Assay

To 1 mL of plant extract, 100 µL of 2 mM ferric chloride was added and incubated at room temperature for 30 min; then, 200 µL of 5 mM ferrozine was added and vortexed at room temperature for 10 min, and absorbance was read at 562 nm. Ascorbic acid at a concentration of 1 mg/mL was used as a standard to determine the antioxidant profile of *J. adhatoda* and *S. cordifolia* [16].

### 2.4. LC-ESI-MS Analysis of Justicia adhatoda

The LC-MS/MS analysis was carried out using Shimadzu LC-MS/MS with C18 column with a scan range of 50–2000 m/z in positive and negative modes with the mobile phase of 0.1% (v/v) formic acid (A) and acetonitrile (B) in gradient method. The injection volume was 0.8 mL/min with dry gas at a flow rate of 10.0 L/min in the negative mode and 8.0 L/min in positive mode at 220°C. For data analysis, different tools such as METLIN, KEGG, and PubChem were used [17].

### 2.5. Molecular Docking

To determine the interactions between the bioactive compounds identified from the LC-MS/MS analysis of *S. cordifolia* [12] and *J. adhatoda* with human beta secretase, –BACE1 (PDB ID: 4 WY1) protein PyRx software was used. The 3D structures of these proteins were retrieved from RCBS Protein Data Bank. To remove other interacting ligand molecules and chains, BioVia_2021 Discovery Studio was used. Polar hydrogen bonds were added by Chimera 1.16 tool, and then, these protein structures were converted to PDBQT using PyRx. The structure of bioactive compounds identified was downloaded in SDF format and charges were minimized and converted using PyRx. Docking was carried out using the AutoDock tool site-specific docking was carried out using a grid box created at specific dimensions. Based on the binding affinity, the best-docked pose of a ligand and the protein was selected and visualized using Discovery Studio in 2D analysis to determine the interacting residues with different bonds [18].

### 2.6. ADMET Profiling of Bioactive Compounds

The identified phytocompounds were selected for in silico ADME-Toxicity (ADMET) and drug-likeness calculations using SwissADME online server (http://www.swissadme.ch/, accessed on 12 April 2023). The following parameters were calculated as hydrogen bond acceptors, hydrogen bond donors, log of n-octanol/water partition coefficient (logP), molecular polar surface area, Veber rule, molecular weight and Lipinski’s rule of five. For ADMET prediction, parameters like blood–brain barrier penetration, hepatotoxicity, plasma membrane binding, aqueous solubility, intestinal absorption and cytochrome P450 (CYP) 2D6 inhibition were determined [19]. To predict the toxicity of various organs and determine the LD_50_ value of the compounds, ProTox II online server was used [20,21].

### 2.7. Molecular Dynamics Simulation

Desmond module was used to examine the stability and intermolecular interactions of target–ligand docked complex at 100 ns. The protein–ligand complex was preprocessed using the protein preparation wizard in the Desmond–maestro interface [22]. The default parameters were used for the protein preparation, bond orders, optimization of filling the missing residues and hydrogen bonds were added. OPLS force field was used after building the simulation grid box and the conformed trajectories were analyzed by root-mean-square deviation and root-mean-square fluctuation plots through radius of gyration and hydrogen bond analysis [23].

### 2.8. Binding Free Energy Calculations of Molecules with BACE-1

The prime MM/GBSA module of the Schrodinger suite with OPLS Force field was used to calculate the binding free energy of the protein–ligand complex based on the following formula:

ΔG_bind_ = ΔE_MM_ + ΔG_solv_ + ΔG_SA_

where, ΔG_bind_ is the binding free energy of the protein–ligand system, ΔG_solv_ represents the GBSA salvation energy difference of the protein–ligand complex and the sum of the solvation energies for the free receptor and free inhibitors. ΔE_MM_ is the minimized energy difference between ligand complex and the sum of the free protein and inhibitor. ΔG_SA_ represented the surface area difference between the complex and the sum of the surface area energy for the unbound ligand-receptor complex. The binding free energy tool and docking score were considered to optimize the selection of the ligands [24,25,26].

## 3. Results

### 3.1. Antioxidant Profiling of Justicia adhatoda and Sida cordifolia

The total phenolic content present in *S. cordifolia* root was found to be highest compared to the leaf and stem part of the plant with a concentration of 154 ± 1.0 and 145.5 ± 1.0 GAE/g, respectively; in *J. adhatoda* leaf extract, the concentration was found to be 137 ± 1.0 GAE/g in the methanolic extract. Total flavonoid content of *J. adhatoda* leaf extract was found to be 40.3 ± 0.3 mg of quercetin per gram and in *S. cordifolia* highest flavonoid content was found in root tissue at a concentration of 567.0 ± 1.0 mg of quercetin per gram compared to root and stem tissues. The radical scavenging activity of the extracts toward DPPH was evaluated spectrophotometrically at 517 nm by the reduction of the free radical change from violet to yellow color by the addition of the methanolic extracts of *J. adhatoda* and *S. cordifolia*. The results obtained were compared to the standard ascorbic acid, where *S. cordifolia* root extract showed the highest scavenging activity of 476.9 ± 3.0 μM of ascorbic acid equivalent compared to the leaf and stem parts. In *J. adhatoda*, the methanolic leaf extract showed the highest concentration of 345 ± 1.0μM of ascorbic acid equivalent. *S. cordifolia* and *J. adhatoda* extracts were used to determine the reducing power of iron from Fe^2+^ to Fe^3+^ at an absorbance of 562 nm. The results showed that *S. cordifolia* root (591.06 ± 0.5 μM ascorbic acid equivalents) extract had the highest iron reducing power compared to other tissues and *J. adhatoda* leaf extract has a reducing power of 154.08 ± 1.8 μM ascorbic acid equivalents.

### 3.2. LC-MS/MS Analysis of Justicia adhatoda L.

Although the spectrophotometric analysis provides information regarding the number of phytochemicals present in them, the exact composition of various phytochemicals present in the extract has not been determined. Thereby, the extracts were subjected to liquid chromatography coupled with mass spectrometry for the detailed profiling of *J. adhatoda* methanolic leaf extract. The list of compounds identified tentatively from the methanolic extract of *J. adhatoda* and *S. cordifolia* is summarized in Table 1 and Table S1, respectively. The compounds in Table S1 were obtained from previously reported data [12].

### 3.3. Molecular Docking

**Figure 1:**
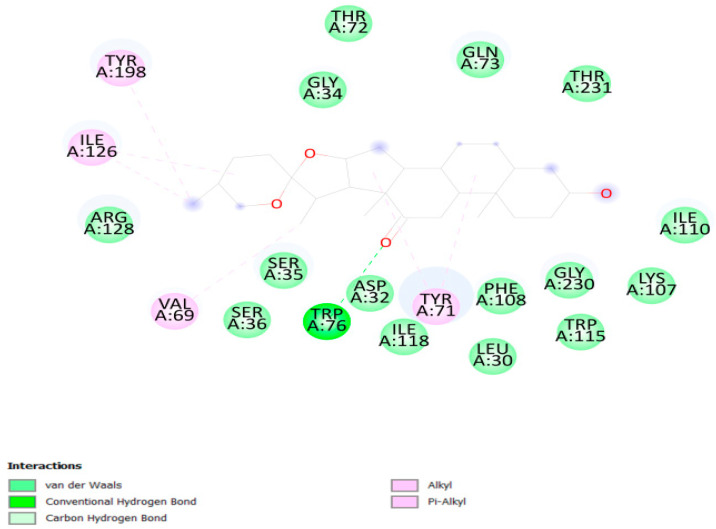
Interacting residues of Hecogenin and Human BACE-1.

A total of 36 compounds were selected from LC-MS/MS analysis of *S. cordifolia* (root, stem and leaf) and *J. adhatoda* (leaf) methanolic extracts. These phytocompounds were docked with the BACE-1 protein, which is the major target for Alzheimer’s disease. The protein–ligand docking was carried out using Vina wizard in PyRx tool for elucidation of binding affinities to the targeted proteins and site-specific docking was done with the active sites predicted by the online server CASTp 3.0 (http://sts.bioe.uic.edu/castp/index.html?1bxw, accessed on 10 April 2023). Hecogenin, which was previously reported in *S. cordifolia* [12], exhibited the lowest binding affinity of −11.3 kcal/Mol against human BACE-1 protein. The list of best binding affinities of the phytocompound and the protein are represented in Table 2. We analyzed the amino acid residues interacting with the ligand and the nature of the forces that they are involved with using the Biovia Discovery Studio Visualizer in 3D and 2D format. The amino acid residues interacting with Hecogenin and Human BACE-1 protein are ARG SER 36, ASP 32, ILE 118, PHE 108, LEU 30, TRP 115, LYS 107, THR 231, THR 72 (Van der waals) and TRP 76 (Conventional hydrogen bonds) (Figure 1).

### 3.4. ADMET Profiling

To predict the absorption, distribution, metabolism and excretion properties of the selected phytocompounds—and to identify their drug-likeness and pharmacokinetic properties—we used SwissADME, an online tool (http://www.swissadme.ch/, accessed on 10 April 2023). Based on the Lipinski rule, we calculated ADME profiling where the molecular weight < 500, TPSA (Topological surface area) < 150, number of hydrogen bond donors < 5, hydrogen bond acceptor < 10 and up-to two violations the Lipinski rule can be accepted for the drug (Table 3) [27]. Except 3-p-Coumaroylquinic acid, ferulic acid O-glucoside, rosmarinic acid and quinic acid all compounds showed good gastro-intestinal absorption (GI absorption). The compounds 3-p-Coumaroylquinic acid, acacetin, apigenin, caffeic acid, catechin, ferulic acid O-glucoside, malic acid, luteolin, quercetin, quinic acid, rosmarinic acid, 3’,4’,7-trihydroxyisoflavanone, atenolol, Dinitrocresol and Trimethoprim predicted that they cannot cross the blood–brain–barrier (BBB). To determine the toxicity, ProTox II (https://tox-new.charite.de/protox_II/index.php?site=compound_input, accessed on 10 April 2023) web server was used and the compounds were mostly predicted to be non-mutagenic, cytotoxic, carcinogenic, immunotoxic and hepatotoxic, except dinitrocresol, loliolide, teniposide and verapamil (Table S2). The compounds having LD_50_ values greater than 2000 mg/kg, demonstrated that they may be safe for in vivo studies as potential medicinal products.

### 3.5. Molecular Simulation

**Figure 2:**
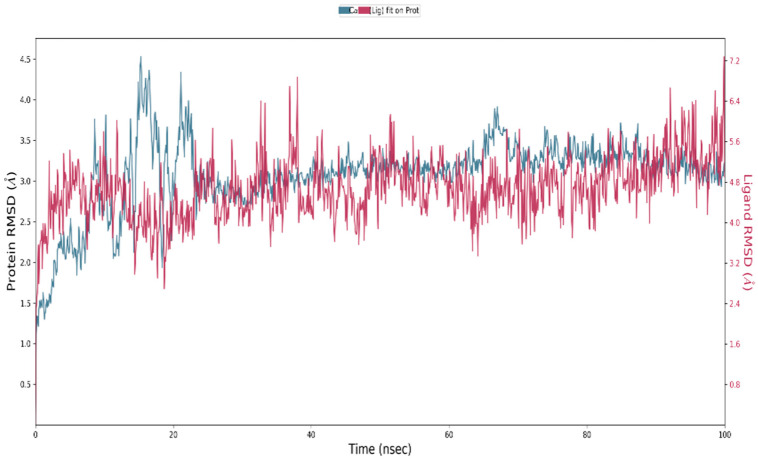
RMSD plot for hecogenin and BACE-1 complex with the RMSD of the protein backbone and the molecular dynamics trajectory of 100 ns.

**Figure 3:**
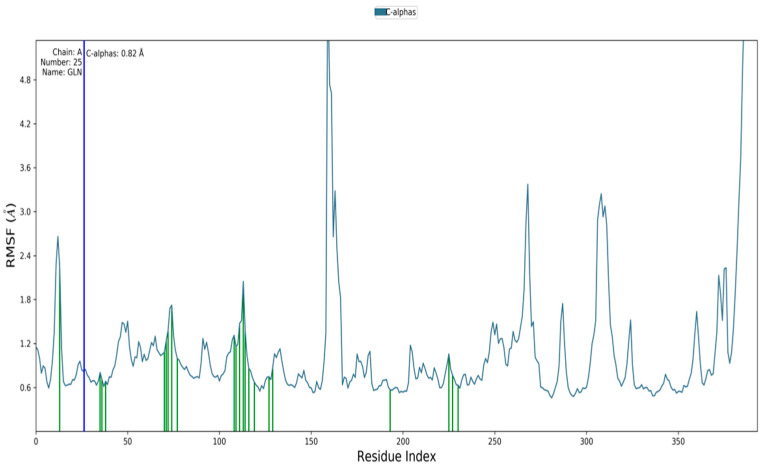
RMSF plot of human BACE-1 protein chain with the ligand bound state.

Molecular dynamics simulation studies were performed to determine the stability between hecogenin and human BACE-1 protein using Desmond tool in the Schrödinger Suite. The results obtained are depicted in Figure 2, where the X-axis represents the time in nanoseconds (ns); in our study, we ran the simulations up to 100 ns. Figure 2 represents the RMSD of BACE-1 protein on the left of Y-axis and the RMSD of hecogenin on the right side of Y-axis. The RMSD value represents the structural conformation of the protein and stability of the ligand–protein complex throughout the simulations. The protein–ligand complex was found to be stable at 30 ns based on the RMSD value, and the protein was found to be stable throughout the simulations of 100 ns which imply that the structural conformation of the receptor protein in the bound–ligand state was stable. To characterize the local changes throughout the BACE-1 protein, root-mean-square fluctuation (RMSF) was used. The peaks indicate areas of protein that fluctuated the most during the simulations. It is observed that the tails N- and C-terminal fluctuate more than other parts of the protein. In Figure 3, the blue line represents the backbone atoms of the protein moiety with the carbon, nitrogen and oxygen atoms that are involved in the peptide formation. The green line represents the side chain atoms of the protein atoms that are extended from the backbone. The grey line represents the overall RMSF values used to calculate the flexibility and fluctuation of the complete protein.

**Figure 4:**
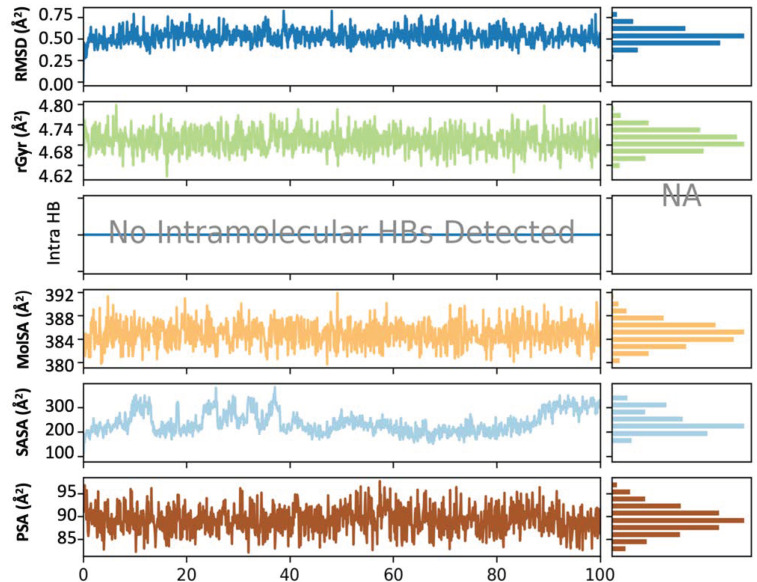
RMSD, radius of gyration (rGyr), intramolecular hydrogen bond (intraHB), molecular surface area (MolSA), solvent accessible surface area (SASA), polar surface area (PSA) of the ligand–protein complex as calculated during the 100 ns of MD Simulation.

**Figure 5:**
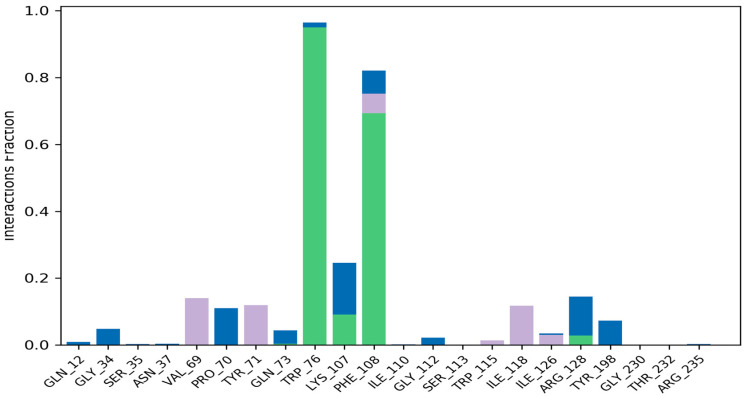
The bar graph represents the interactions between BACE-1 and hecogenin throughout the simulations of 100 ns in different colors signifying the type of interactions between the amino acids and the ligand.

The alpha helices, beta strands of the secondary structures along with the loop regions are more stable compared to the unstructured part of the protein. Figure 4 represents the RMSD, polar surface area (PSA), molecular surface area (MolSA), radius of gyration (rGyr) and solvent accessible surface area (SASA) of the protein–ligand complex in 100 ns long simulation. Supplementary Figure S1 represents the formation and interactions of hydrogen bonds with Phe 404 and 108. The interaction between the protein–ligand complexes throughout the molecular dynamic simulations has been represented in Figure 5. A timeline representation of the interacting residues of the protein with ligand molecule is depicted in the Supplementary Figure S2.

Prime molecular mechanics generalized born surface area (MM/GBSA) was used to determine the strength of the complex by calculating the binding free energy. Based on the binding free energy calculated, hecogenin showed a significantly lower binding energy of −57.7393 kcal/mol compared to naringin 6″-malonate (−50.3731 kcal/mol) with BACE-1. Therefore, the molecular docking, dynamics and simulation studies along with binding free energy suggest that hecogenin steadily binds with BACE-1 (Table 4).

## 4. Discussion

*S. cordifolia* and *J. adhatoda* are two medicinal plants with innumerable medicinal properties. In the present study, we investigated the methanolic extracts of both the plants and divulged the presence of various groups of phytochemicals such as terpenoids, flavonoids, alkaloids, steroids and saponins. In a recent study, the radical scavenging activity of the *S. cordifolia* extract is due to the presence of polyphenolic compounds, acting as an antioxidant defense mechanism [28]. The results obtained from our study revealed that among three tissues (root, leaf and stem) of *S. cordifolia*, only the root part of the plant was found to possess a good antioxidant property due to its radical scavenging activity, total flavonoid and total phenolic contents. The results are in correspondence to the earlier study which concluded that amongst eight different species of *Sida* genus, only *S. cordifolia* was found to comparatively possess a good antioxidant and polyphenolic content [29]. Studies on the chemotherapeutic properties of the plant extract have opened a new door for less toxic and more effective medications by using natural compounds to treat various diseases such as cancer. Previous studies on the leaf extract of *J. adhatoda* on MCF-7 (breast cancer) cell line were found to promote cell death and inhibition of colony formation in anchorage-independent manner [30,31]. Based on the therapeutic potential of phytocompounds as reported in *S. cordifolia* and *J. adhatoda*, molecular docking and simulation studies revealed its binding affinity and interaction with the target protein of AD.

BACE-1 has become a main target for diminution of AD as they are found to elevate the levels of amyloidal production in patients with AD compared to normal aging brain [32,33,34]. Previous studies suggest that nutraceuticals act as a neuroprotective as well as providing an anti-viral and anti-cancer therapeutic approach. A study on nutraceutical 4-(3, 4-dihydroxyphenyl)-2-hydroxy-1H-phenalen-1-one proved its binding affinity of −8.9 kcal/mol, the molecular dynamics simulation was run for 150 ns identified that BACE-1 protein structural motifs were involved in the interaction with the compound [35]. This study revealed higher binding affinity of −11.3 kcal/mol with hecogenin-BACE-1 protein. The molecular dynamics simulation studies of the protein–ligand resulted in stable conformational complex. In another study, BACE-1 protein was docked with phytocompounds isolated from *Rosmarinus officinalis*, which predicted that polyphenol has a binding energy of −7.45 and was validated with stabilized orientation in molecular dynamics analysis [36].

Hecogenin is a steroidal saponin used in pharmaceutical companies as a precursor for the synthesis of steroidal hormones. A study conducted by Jullyana et al. suggested that the compound hecogenin has an anti-inflammatory property which inhibits hyperalgesia development when induced with carrageenan, reducing dopamine and TNF-alpha production. The compound was concluded to block the neural transmission of pain in the spinal cord region by inhibiting cytokine mechanism [37,38]. Vascinone, an alkaloid derived from *J. adhatoda*, was studied to investigate the neuroprotective effect in parquet-mimic Parkinson’s disease model in SH-SY5Y cells. The study revealed that antioxidant balance was maintained by the enzymes which scavenged the reactive oxygen species [39].

The pandemic caused by the outbreak of the SARS virus has been treated with some phytocompounds derived from medicinal plants. In one such study, myricetin has proven to possess anti-viral, anti-inflammatory and anti-bacterial activity, and also, provides protection against cardiovascular and neurological diseases. The compound was also found to have good ADMET properties which provides it with further validation as an anti-viral agent in treating SARS-CoV-2 infection [40,41,42]. In a study conducted by Jayaraman et al., they concluded that ethambutol, pyrazinamide, vasicoline and stigmasterol have anti-diabetic effects when docked against Glycogen synthase kinase-3; these compounds were derived from *J. adhatoda* [43].

Molecular dynamic simulations provide a stable binding model of the receptor and ligand. Along with studying the effect of surrounding explicit water molecules—though they are time consuming and incur a high computational cost—they are used as a paramount for drug designing [44,45,46]. The constant increase in RMSD values with time indicated that the protein serves from its native form. The higher RMSF in the study proves that strong hydrogen bonds with polar amino acids have the maximum stability.

Tox II and SwissADME analysis shows the absorption profiles, permeability and toxicity of the drug in different organs to demonstrate its potential to be used as a commercially formulated drug. This virtual screening tool elucidated that out of the 54 compounds identified through LC-MS/MS analysis of both the plants, 21 compounds were found to have a potential to act as a drug for further research. The integration of structure based virtual screening is the most robust and promising in silico tool for drug designing. Though this kind of virtual screening leads us to a faster and more reliable way to drug discovery, it is also a way to yield false positive results, and therefore, it should be followed by biological approach [47]. Though many synthetic drugs have been commonly used to treat AD and cancers, phytocompounds can act as a source of novel drugs to prevent the side effects of the commercialized products currently available for treatment. Our results support further avenue for clinical and in vivo trails of phytocompounds to target the proteins to treat AD.

## 5. Conclusions

The present investigation utilized LC-MS/MS to identify numerous biologically active substances from distinct tissues of *J. adhatoda* and *S. cordifolia* for the first time. The medicinal benefits are attributed to bioactive compounds found in methanolic extracts of both the plants. The study suggests that these extracts have a prospect for antioxidant activity. In molecular docking studies, the naturally occurring compounds hecogenin, naringin 6” malonate, procyanidin B2 and kaempferol-dirhamnoside illustrated promising binding affinity towards the BACE-1 protein receptor for Alzheimer’s disease. Hecogenin had the best docking against BACE-1 among all other compounds. The ligand–protein complex was then subjected to molecular simulation and dynamics to analyze stability, RMSD and RMSF values. Furthermore, ADMET profiling of hecogenin revealed the strongest drug-likeness attribute among all other compounds. With extensive in vitro and in vivo investigations, it could potentially be used as a potent therapeutic, facilitating in the discovery of herbal-based formulations for safe and effective medication.

## Figures and Tables

**Table 1:** List of phytocompounds identified from LC-MS/MS analysis of *Justicia adhatoda*.

S.No.	RT	Compound	Mode	Molecular Formula	m/z	Adduct
1	13.43	Verapamil	Negative	C_27_H_38_O_4_	455.291, 456.298, 303.206	[M − H]^−^
2	46.84	Cynarine	Negative	C_25_H_24_O_12_	515.123, 353.10, 191.021	[M − H]^−^
3	53.5	Coumaroyl Hexoside	Negative	C_15_H_18_O_8_	163.038, 145.012, 119.047	[M − H]^−^
4	88.7	Teniposide	Negative	C_32_H_32_O_13_S	701.100, 113.123, 655.126	[M − H]^−^
5	93.59	Shingomyelin	Negative	C_39_H_79_N_2_O_6_P	687.544, 301.218, 762.510	[M + Hac-H]^−^
6	8.2	2’-Deoxyadenosine	Positive	C_10_H_13_N_15_O_3_	252.399, 113.324, 123.238	[M + H]^+^
7	13.19	Atenolol acid	Positive	C_14_H_21_NO_4_	269.156, 270.1558, 226.105	[M + H]^+^
8	14.06	Glutamylphenylalanine	Positive	C_14_H_18_N_2_O_5_	278.178, 277.120	[M + H]^+^
9	26.83	9,10-EODE	Positive	-	-	
10	30.91	trans-4-hydroxycinnamic acid	Positive	C_9_H_8_O_3_	147.043, 165.054, 91.053	[M + H]^+^
11	39.84	Chlorogenate	Positive	C_16_H_18_O_9_	135.043, 163.037, 145.027	[M + H]^+^
12	56.87	Dinitrocresol	Positive	C_7_H_6_N_2_O_5_	197.020, 137.0244, 167.022	[M + H]^+^
13	61.82	Ritonavir	Positive	C_37_H_48_N_6_O_5_S_2_	721.325, 296.144, 426.187	[M + H]^+^
14	63.24	Quercetin-3-O-arabinoglucoside	Positive	C_26_H_28_O_16_	303.049, 304.052,145.0474	[M + H]^+^
15	66.51	2-Napthalenesulfonic acid	Positive	C_10_H_9_NO_3_S	223.001	[M + H]^+^
16	69.1	Rutin	Positive	C_27_H_30_O_16_	609.148, 610.151	[M + H]^+^
17	79.1	Trimethoprim	Positive	C_14_H_18_N_4_O_3_	291.146, 123.066	[M + H]^+^

**Table 2:** List of ligands with good binding affinity.

Macromolecule	Ligand	Binding Affinity (kcal/mol)
Human BACE-1 protein	Hecogenin	−11.3
	Naringin 6″-malonate	−10.2
	Procyanidin B2	−10
	Kaempferol-dirhamnoside	−9.9

**Table 3:** List of compounds with the best ADME profiling.

Compound	Molecular Weight (g/mol)	Num. H-Bond Acceptor	Num. H-Bond Donor	TPSA (Å^2^)	GI Absorption	BBB Permeant	Lipinski
3-p-Coumaroylquinic acid	338.31 g/mol	8	5	144.52	Low	No	Yes; 0 violation
Acacetin	284.26 g/mol	5	2	79.9	High	No	Yes; 0 violation
Apigenin	270.24 g/mol	5	3	90.9	high	No	Yes; 0 violation
Caffeic acid	180.16 g/mol	4	3	77.76	High	No	Yes; 0 violation
Cinnamaldehyde	132.16 g/mol	1	0	17.07	High	yes	Yes; 0 violation
Cinnamic acid	148.16 g/mol	2	1	37.3	High	yes	Yes; 0 violation
Catechin	290.27 g/mol	6	5	110.38	High	no	Yes: 0 violation
Ferulic acid O-glucoside	356.32 g/mol	9	5	145.91	Low	no	Yes; 0 violation
Hecogenin	430.62 g/mol	4	1	55.76	High	yes	Yes; 0 violation
Loliolide	196.24 g/mol	3	1	46.53	High	yes	Yes; 0 violation
Malic acid	134.09 g/mol	5	3	94.83	High	no	Yes; 0 violation
Luteolin	286.24 g/mol	6	4	111.13	High	no	Yes; 0 violation
Quercetin	302.24 g/mol	7	5	131.36	High	no	Yes; 0 violation
Quinic acid	192.17 g/mol	6	5	118.22	Low	no	Yes; 0 violation
Rosmarinic acid	360.31 g/mol	8	5	144.52	Low	no	Yes; 0 violation
3’,4’,7-trihydroxyisoflavanone	272.25 g/mol	5	3	86.99	High	no	Yes; 0 violation
Verapamil	454.60 g/mol	6	0	63.95	High	yes	Yes; 0 violation
Atenolol	266.34 g/mol	4	3	84.58	High	no	Yes; 0 violation
4-Hydroxycinnamic acid	164.16 g/mol	3	2	57.53	High	yes	Yes; 0 violation
Dinitrocresol	198.13 g/mol	5	1	111.87	High	no	Yes; 0 violation
Trimethoprim	290.32 g/mol	5	2	105.51	High	no	Yes; 0 violation

**Table 4:** Relative binding free energy of the top two phytocompounds with BACE-1 protein.

Compound	MMGBSA-dG-Binding Energy (kcal/mol)	MMGBSA-dG-Bind in Coulomb (kcal/mol)	MMGBSA-dG-Bind(NS) (kcal/mol)	MMGBSA-dG-Bind(NS)-Coulomb (kcal/mol)
Hecogenin	−57.7393	−7.53358	−60.3922	−7.43166
Naringin6″-malonate	−50.3731	−15.0102	−52.1919	−15.2455

## Data Availability

No data is available due to privacy.

## Supplementary Materials

The following supporting information can be downloaded at: https://www.mdpi.com/article/10.3390/metabo13060758/s1, Figure S1: Interactions of hecogenin with BACE-1; Figure S2: Protein RMSF; Figure S3: The number of ligand contacts with protein and the amino acid sites; Table S1: List of compounds identified from LCMS/MS analysis of *Sida cordifolia*; Table S2: Toxicity profiling of Phytocompounds.
