# Supplementary material for: Hecogenin a Plant Derived Small Molecule as an Antagonist to BACE-1: A Potential Target for Neurodegenerative Disorders
Source: Metabolites. 2023 Jun 16;13(6):758. doi: 10.3390/metabo13060758 (PMC10303843; doi:10.3390/metabo13060758)
Supplement: Supplementary file 1 [file metabolites-13-00758-s001.zip › metabolites-2407166-supplementary.pdf]

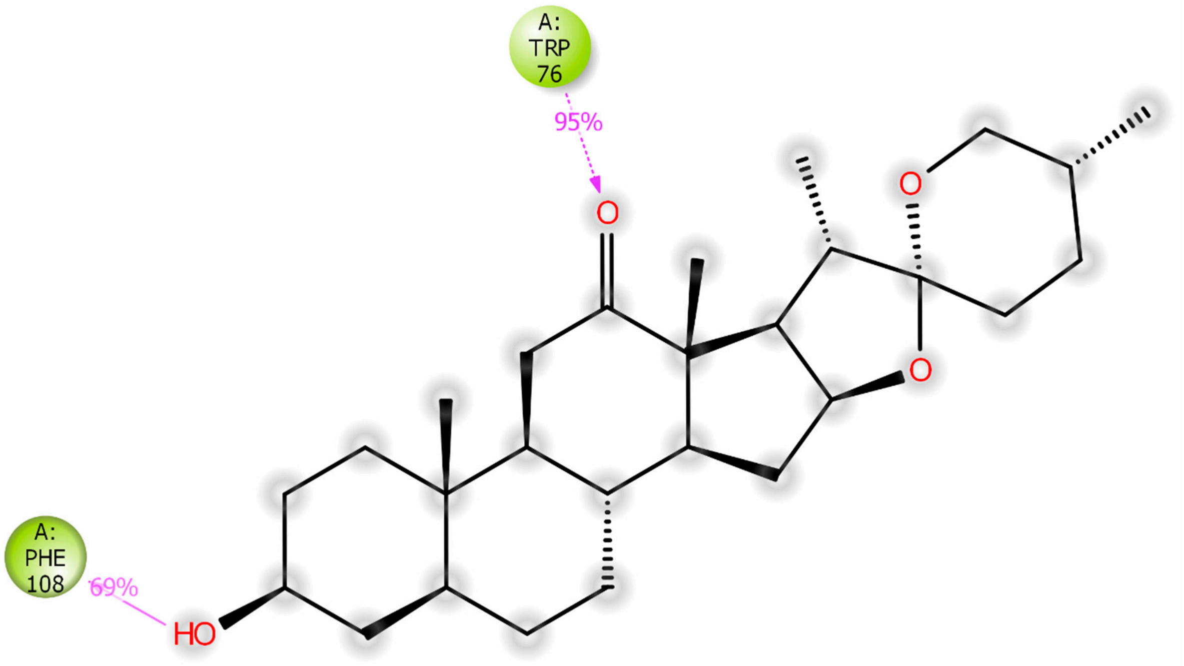

Supplementary Figure S1: Interactions of hecogenin with BACE-1

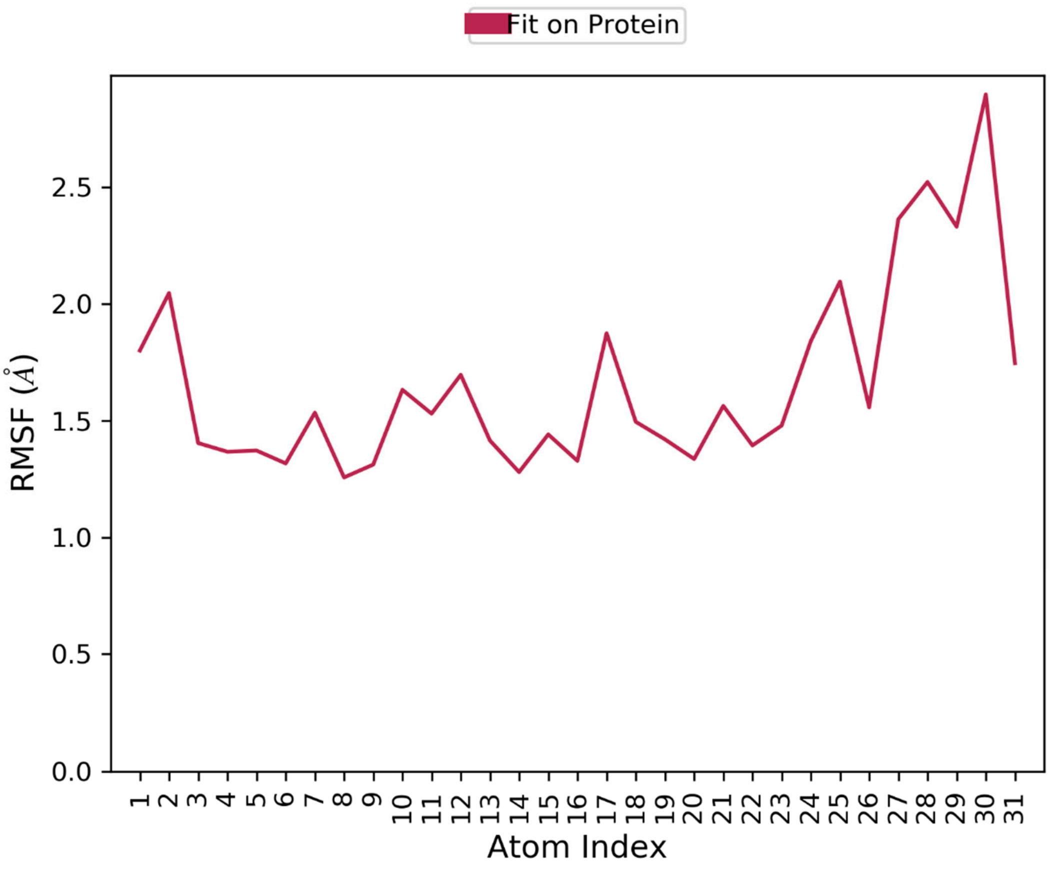

Supplementary Figure S2: Protein RMSF

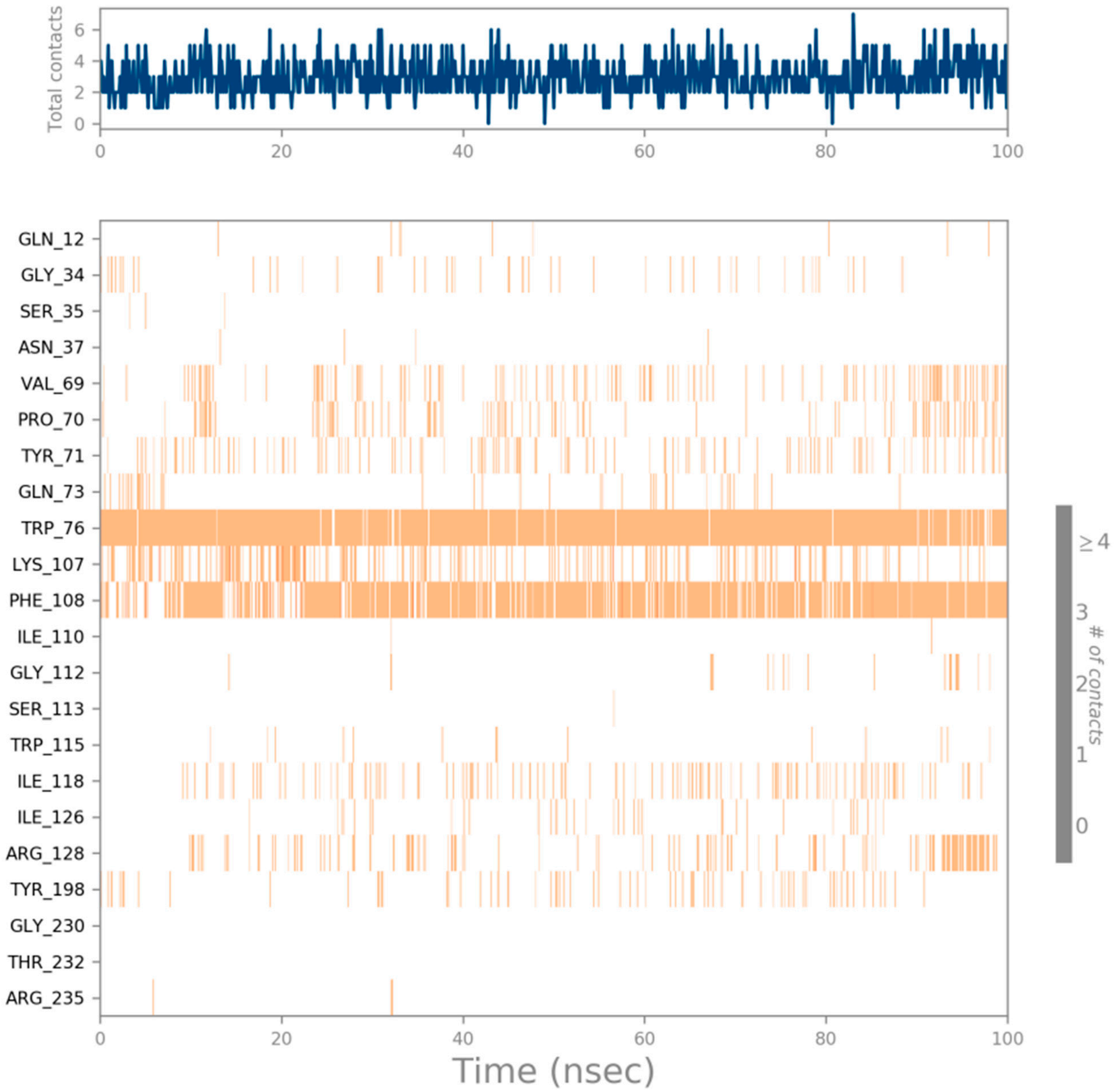

Supplementary Figure S3: The number of ligand contacts with protein and the amino acid sites
